# Valproic acid exhibits anti-tumor activity selectively against EGFR/ErbB2/ErbB3-coexpressing pancreatic cancer via induction of ErbB family members-targeting microRNAs

**DOI:** 10.1186/s13046-019-1160-9

**Published:** 2019-04-08

**Authors:** Tingting Lin, Qun Ren, Weimin Zuo, Ruxue Jia, Linhui Xie, Rong Lin, Hu Zhao, Jin Chen, Yan Lei, Ping Wang, Huiyue Dong, Lianghu Huang, Jinquan Cai, Yonghai Peng, Zongyang Yu, Jianming Tan, Shuiliang Wang

**Affiliations:** 1 Department of Urology, The 900th Hospital of the Joint Logistics Team (the Former Fuzhou General Hospital), Fujian Medical University, Fuzhou 350025, China. 156 Xi’er Huan Bei Road, Fuzhou, 350025 Fujian Province China; 2 Fujian Key Laboratory of Transplant Biology, Affiliated Dongfang Hospital, Xiamen University School of Medicine, Fuzhou, 350025 Fujian Province China; 3 Department of Medical Oncology, First Hospital of Sanming, Sanming, 365000 Fujian Province China; 4 Department of Medical Ultrasound, Guangzhou First Peoples’s Hospital, Guangzhou Medical University, Guangdong Province, Guangzhou, 510180 China; 5 Department of Clinical Medicine, Fujian Health Vocational and Technical College, Fuzhou, 350101 Fujian Province China; 6 Department of Medical Oncology, The 900th Hospital of the Joint Logistics Team (the Former Fuzhou General Hospital), Fujian Medical University, Fuzhou, 350025 Fujian Province China

**Keywords:** Valproic acid, Pancreatic cancer, ErbB family members, microRNAs

## Abstract

**Background:**

Deregulated ErbB signaling plays an important role in tumorigenesis of pancreatic cancer. However, patients with pancreatic cancer benefit little from current existed therapies targeting the ErbB signaling. Here, we explore the potential anti-tumor activity of Valproic acid against pancreatic cancer via targeting ErbB family members.

**Methods:**

Cell viability assay and apoptosis evaluation were carried out to determine the efficacy of VPA on pancreatic cancer cells. Western blot analyses were performed to determine the expression and activation of proteins. Apoptosis enzyme-linked immunosorbent assay was used to quantify cytoplasmic histone associated DNA fragments. Lentiviral expression system was used to introduce overexpression of exogeneous genes or gene-targeting short hairpin RNAs (shRNAs). qRT-PCR was carried out to analyze the mRNAs and miRNAs expression levels. Tumor xenograft model was established to evaluate the in vivo anti-pancreatic cancer activity of VPA.

**Results:**

VPA preferentially inhibited cell proliferation/survival of, and induced apoptosis in EGFR/ErbB2/ErbB3-coexpressing pancreatic cancer cells within its clinically achievable range [40~100 mg/L (0.24~0.6 mmol/L)]. Mechanistic investigations revealed that VPA treatment resulted in simultaneous significant down-regulation of EGFR, ErbB2, and ErbB3 in pancreatic cancer cells likely via induction of ErbB family members-targeting microRNAs. Moreover, the anti-pancreatic cancer activity of VPA was further validated in tumor xenograft model.

**Conclusions:**

Our data strongly suggest that VPA may be added to the treatment regimens for pancreatic cancer patients with co-overexpression of the ErbB family members.

**Electronic supplementary material:**

The online version of this article (10.1186/s13046-019-1160-9) contains supplementary material, which is available to authorized users.

## Background

The worldwide mortality of pancreatic cancer ranks eighth and ninth among all cancers in male and female, respectively [1]. Owing to lack of clinically validated screening methods for pancreatic cancer in the curative stage, fewer than 20% of patients will be diagnosed with localized tumors and surgery remains the only option for cure [2]. Thus far the overall 5-year survival rate among patients with pancreatic cancer is < 5% [3, 4].

Mainly arising from pancreatic ductal cells, pancreatic cancer could also develop from acinar cells as evidenced [5–7]. Extensively genetic studies have revealed that during the progression of three broad stages of pancreatic cancer, acquired somatic mutations in oncogenes and tumor suppressor genes accumulate and account for initiation and aggressive development of this malignant disease. These mutations occur most frequently in *KRAS*, *CDKN2A*, *TP53* and *SMAD4* [8–10]. Activating mutations of the *KRAS* oncogene, which encodes a member of the RAS family of GTP-binding proteins, are the most common genetic abnormality presenting in approximately 95% of pancreatic tumors analyzed [11, 12]. In addition, wild-type KRAS is also normally activated in response to the binding of extracellular signals such as growth factors to receptor tyrosine kinases (RTKs) [13]. Among all subfamilies of RTKs, the ErbB family members consisting of the epidermal growth factor receptor EGFR (ErbB1), HER2 (ErbB2), HER3 (ErbB3), and HER4 (ErbB4) play important role in the initiation and maintenance of a variety of human cancers, including pancreatic cancer [14, 15]. Accumulated evidence shows that the ErbB receptors overexpress in approximately 60% of pancreatic cancers [16]. Collectively, deregulated RTKs/RAS/RAF/MEK/MAPK signaling pathway is undoubtedly important for pancreatic cancer biology, and extensive efforts have been taken to target this pathway for systemic therapy [17–20].

In addition to gene amplification and mutation, alterations in chromatin structure by histone modification and/or DNA methylation also play a vital role in transcriptional regulation of oncogene or tumor suppressors in human cancers [21]. Thus, epigenetic targeting is emerging as a promising therapeutic strategy for cancer treatment. Histone deacetylases (HDACs), whose deregulation is evidenced to play an important role in aberrant gene expression in tumorigenesis, have long been recognized as druggable targets [22]. We have previously found that the class I HDAC inhibitor (HDACi), entinostat (also known as MS-275 or SNDX-275) specifically enhanced expression of miR-125a, miR-125b, and miR-205, which acted in concert to downregulate ErbB2 and ErbB3 in ErbB2-overexpressing breast cancer cells [23, 24]. In our attempt to identify novel strategy targeting RTKs signaling in pancreatic cancer, we noticed that Valproic acid (VPA), a safely used anti-convulsant drug in the treatment of epilepsy and other seizure disorders, was reported to exert potent anti-tumor activity in a number of cancers owing to its HDACi capability [25]. However, the underlying mechanism of VPA against human cancers remains poorly understood. In our current study, we have explored the potential therapeutic efficacy of VPA on pancreatic cancer using both an in vitro cell culture system and an in vivo tumor xenograft model. The molecular basis of VPA-mediated anti- pancreatic cancer activity was also elucidated.

## Methods

### Reagents and antibodies

Valproic acid and LY294002 were purchased from Sigma-Aldrich (St. Louis, MO, USA) and dissolved in ddH_2_O or dimethyl sulfoxide (DMSO) to make a stock solution at 500 mmol/L or 20 mmol/L, respectively. All the stock solutions were stored at − 20 °C. Recombinant human NRG-1 protein ab50227 was product from abcam (Cambridge, MA, USA).

MISSION® Non-target shRNA, which does not target human and mouse genes, control vector (pLKO.1-ConshRNA), and pLKO.1 containing human *erbB3* shRNA (pLKO.1-ErbB3shRNA) were purchased from Sigma. The packaging plasmids psPAX2 and pMD2.G for lentiviral expression vector were from Addgene Inc. (Cambridge, MA, USA).

Antibodies were obtained as follows: EGFR, ErbB2, ErbB3, PARP, Cleaved Caspase-3 (Asp175) (5A1E), P-MAPK (E10), MAPK, P-Akt (Ser473), Akt, STAT3, P-STAT3 (Tyr705), p21, Cyclin D1, RAS, Ki67 (Cell Signaling Technology, Inc., Beverly, MA, USA); β-actin (AC-75) (Sigma). All other reagents were purchased from Sigma unless otherwise specified.

### Cells and cell culture

Human pancreatic adenocarcinoma cell lines HPAF-II, MPanc96, MiaPaca-2, and Panc-1 were purchased from ATCC (Manassas, VA, USA) and maintained in RPMI1640 medium supplemented with 10% fetal bovine serum (FBS). HEK293T human embryonic kidney cells were maintained in DMEM/F12 medium containing 10% FBS. All cell lines were cultured in a 37 °C humidified atmosphere containing 95% air and 5% CO_2_ and were split twice a week.

### Cell viability assay

The CellTiter96AQ cell proliferation kit (Promega, WI, USA) was used to determine cell viability as we previously described [26]. For cell staining assays, human pancreatic cancer cells HPAF-II, MPanc96, MiaPaca-2, and Panc-1 were plated onto 24-well plates and incubated at 37 °C with 5% CO2. After 24 h, the culture medium was replaced with 700 μl of medium containing 0.5% FBS or the same medium containing indicated concentrations of VPA. Cells were incubated in a 37 °C humidified atmosphere containing 95% air and 5% CO_2_ for 72 h. The percentages of surviving cells from each group relative to controls, defined as 100% survival, was determined by reduction of MTS following by staining with 0.5% crystal violet for visualization of viable cells.

### Western blotting analysis and quantification of apoptosis

Protein expression and activation were determined by western blotting analysis as previously described [27]. In brief, equal amounts of cell lysates in a buffer were boiled in sodium dodecyl sulfate sample buffer, resolved by sodium dodecyl sulfate polyacrylamide gel electrophoresis and western blotted with specific antibodies directed against EGFR, ErbB2, and ErbB3, PARP, Cleaved Caspase-3 (C-Casp-3), P-Akt, Akt, P-MAPK, MAPK, P-STAT3, STAT3, RAS, or β-actin, as described in the figure legends. For quantification of apoptosis, an apoptosis enzyme-linked immunosorbent assay kit (Roche Diagnostics Corp., Indianapolis, IN, USA) was used to quantitatively measure cytoplasmic histone associated DNA fragments (mononucleosomes and oligonucleosomes) as previously reported [27]. In addition, cell apoptosis was also detected with flow cytometry assay. Briefly, cells upon treatment with indicated concentration of VPA for 24 h or 48 h were harvested and co-stained with propidiumiodide (PI) and annexin V-fluorescein isothiocyanate (annexin V-FITC). The cells were finally analyzed with a FACSort flow cytometer (Becton-Dickinson, Franklin, NJ, USA). Data were acquired after analysis of at least 10,000 events.

### Construction of lentiviral expression vector pLEX-erbB2 and production of lentivirus

The coding sequence of human *ErbB2* was amplified from pDsRed-erbB2 by PCR with the following forward primer: 5′-ATAGCGGCCGCATGGAGCTGGCGGCCTTGT-3′ and reverse primer: 5′-GCGACGCGTTCACACTGGCACGTCCAGACC-3′. The amplified fragments were inserted into the lentiviral vector pLEX-MCS (Open Biosystem, Huntsville, AL, USA). After sequencing verification, the recombinant was nominated as pLEX-erbB2. Lentiviral production was performed as described [28].

### Analysis of mRNA and miRNA expression with real-time quantitative reverse transcriptase PCR (qRT-PCR)

Total RNA was prepared using TRIZOL reagent (Invitrogen, Carlsbad, CA, USA). First-strand cDNA was generated using High-Capacity cDNA Reverse Transcription Kit (Applied Biosystems, Carlsbad, CA, USA) following the manufacturer’s instructions. The analysis of human *EGFR*, *ErbB2* and *ErbB3* mRNA expression was examined by conventional RT-PCR as we had described previously [24]. To quantify the mRNA levels, qRT-PCR was performed using the FastStart Universal SYBR Green Master Mixes (Roche) by a 7900HT Fast Real-Time PCR system (Applied Biosystems). The expression of *β-actin* was used as an internal control for both conventional RT-PCR and qRT-PCR. The relative mRNA levels were calculated using the comparative Ct method (ΔΔCt). Sequences of specific primers used are listed in Additional file 1: Table S1.

The expression of mature miR-133a, miR-133b, miR-125a, miR-125b, miR-205, and RNU6B was measured by qRT-PCR using TaqMan Assays (Applied Biosystems) as described previously [24]. The relative miRNA levels were calculated using the comparative Ct method (ΔΔCt).

### Tumor xenograft model

Athymic nu/nu mice (Shanghai SLAC Laboratory Animal Co. Ltd., Shanghai, China) were maintained in accordance with the Institutional Animal Care and Use Committee (IACUC) procedures and guidelines approved by the Affiliated Dongfang Hospital of Xiamen University School of Medicine IACUC. Five × 10^6^ HPAF-II or MPanc96 cells were suspended in 50 μL of PBS, mixed with Matrigel (BD Biosciences) and injected subcutaneously into the flanks of 5-week-old female mice. Tumor formation was assessed by palpation and measured with fine calipers three times a week. Tumor volume was calculated by the formula: Volume = (Length × Width^2^)/2, which was statistically analyzed as we described previously [28]. When tumors reach ~ 150 mm^3^, mice were randomly assigned into two groups (*n* = 3): 1) control mice received intraperitoneal (i.p.) injection of 100 μl of PBS; 2) mice received i.p. injection of VPA (500 mg/kg) in 100 μl PBS daily for consecutive 12 days. At the end of study, mice were euthanized according to approved protocol. Tumors were excised and subjected to immunohistochemistry analyses and qRT-PCR measurement of miRNAs expression.

### Immunohistochemistry (IHC)

IHC was performed as described previously [28]. The specificity of all antibodies - EGFR (Cell Signaling Technology; rabbit polyclonal; cat#2232; dilution 1:100 in TBST + 1% BSA *w*/*v*), ErbB2 (EMD Chemicals; mouse monoclonal 96G; cat#OP14T; dilution 1:500 in TBST + 1% BSA w/v), ErbB3 (Spring Bioscience, Pleasanton, CA, USA; rabbit monoclonal SP71; cat# M3710; dilution 1:200 in TBST + 1% BSA w/v), Ki-67 (rabbit monoclonal SP6; dilution 1:500 in TBST + 1% BSA), cleaved caspase-3 (rabbit polyclonal; 1:1000) - has been confirmed by both positive and negative controls. Antibody complexes were visualized with IP Flex DAB (Biocare). All sections were counterstained in Mayer’s hematoxylin, nuclei blued in 1% ammonium hydroxide, cleared in xylene and cover glass mounted by synthetic resin.

### Statistical analysis

All results were confirmed by at least three independent experiments. Data are presented as mean ± SD. “Tow” sided Student’s *t*-tests were used for comparisons of means of quantitative data between groups. A *P*-value < 0.05 was deemed statistically significant. All statistical analyses were conducted with the software StatView v5.1 from SAS Institute Inc. (Cary, NC).

## Results

### VPA preferentially inhibits cell proliferation/survival of EGFR/ErbB2/ErbB3-coexpressing pancreatic cancer cells in vitro

**Fig. 1:**
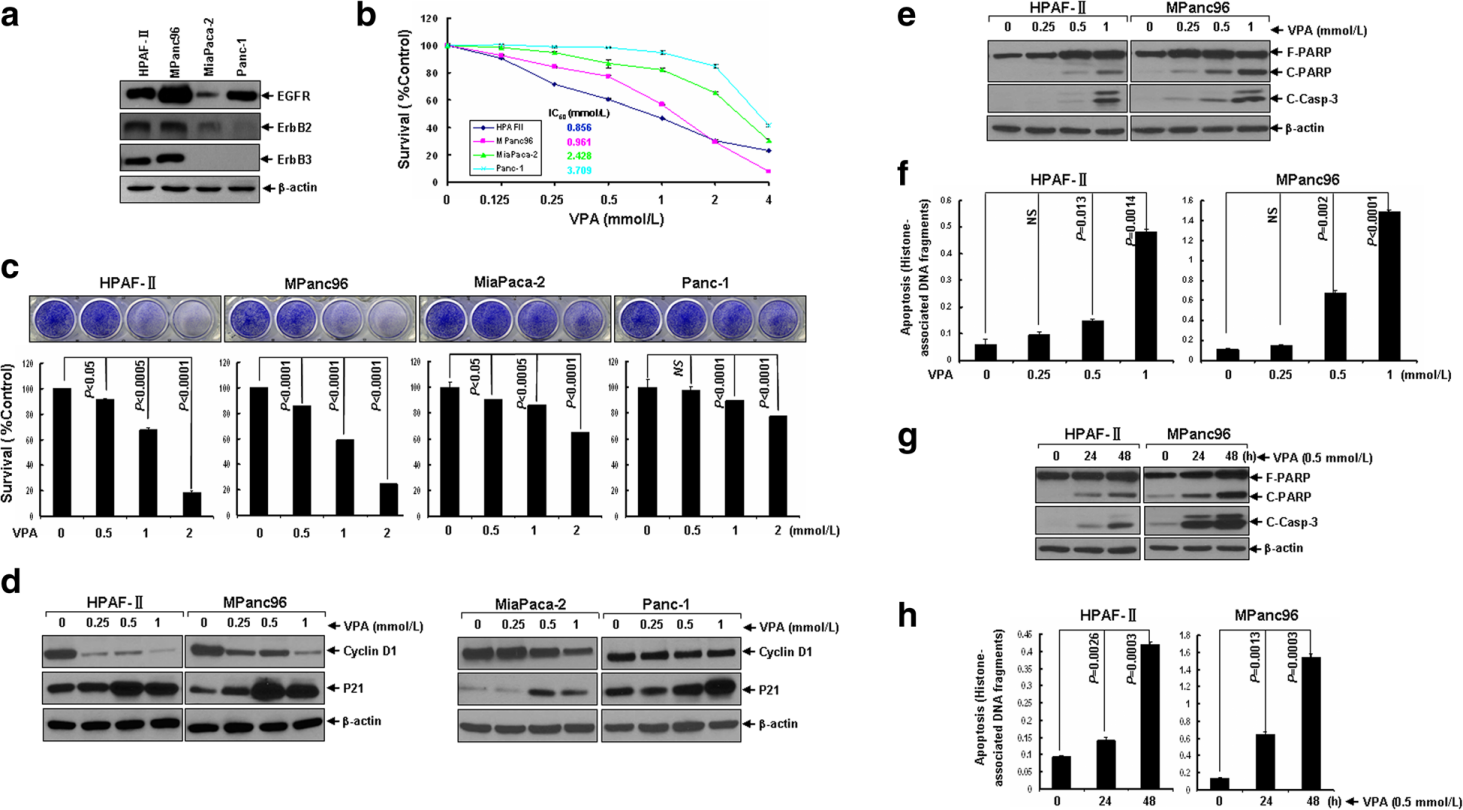
VPA selectively inhibits proliferation/survival of EGFR/ErbB2/ErbB3-coexpressing pancreatic cancer cells. **a** Western blotting analysis of the expression of EGFR, ErbB2, and ErbB3 in pancreatic cancer cells. **b** Human pancreatic cancer cells were plated onto 96-well plates with complete culture medium (RPMI1640, 10% FBS). After 24 h, the medium was replaced with control medium (fresh RPMI1640, 0.5% FBS) or same medium containing indicated concentrations of VPA for another 72 h incubation. The percentages of surviving cells from each treatment to controls, defined as 100% survival, were determined by reduction of MTS. **c** Pancreatic cancer cells were grown in triplicates in the absence or presence of indicated concentrations of VPA for 72 h. The percentages of surviving cells from each group relative to controls, defined as 100% survival, was determined by reduction of MTS following by staining with 0.5% crystal violet for visualization of viable cells. *Bars*, S.D. All data show the representative of three independent experiments. **d** HPAF-II and MPanc96 cells were treated with different concentrations of VPA as indicated for 24 h. Cells were harvested and subjected to western blot analyses with specific antibodies directed against Cyclin D1, P21 or β-actin. **e**, **f** HPAF-II and MPanc96 cells untreated or treated with different concentrations of VPA as indicated for 24 h were harvested and subjected to western blot analyses with specific antibodies directed against PARP, Cleaved Caspase-3 (C-Casp-3) or β-actin (**e**), or apoptotic ELISA (**f**). **g**, **h** HPAF-II and MPanc96 cells untreated or treated with VPA (0.5 mmol/L) for 24 h or 48 h were harvested and subjected to western blot analyses with specific antibodies directed against PARP, C-Casp-3 or β-actin (**g**), or apoptotic ELISA (**h**). *Bars*, S.D. All data show the representative of three independent experiments

To explore whether VPA might show any therapeutic effect on pancreatic cancer, we investigated its anti-proliferative/anti-survival activities in the EGFR/ErbB2/ErbB3-coexpressing (HPAF-II, MPanc96) and ErbB3-negative (MiaPaca-2, Panc-1) pancreatic cancer cell lines (Fig. 1a). We found that VPA inhibited proliferation/survival of all cells in a dose-dependent manner. The EGFR/ErbB2/ErbB3-coexpressing cells were much more sensitive than ErbB3-negative cells to VPA-induced growth inhibition (Fig. 1b). The IC_50_ values were 0.856 mmol/L and 0.961 mmol/L for HPAF-II and MPanc96 cells, and 2.428 mmol/L and 3.709 mmol/L for MiaPaca-2 and Panc-1 cells, respectively. The inhibitory effect of VPA on proliferation/survival of pancreatic cancer cells was further supported by cell staining with crystal violet showing that the survived cells decreased significantly while treating with increased dose of VPA (Fig. 1c). In an effort to investigate the underlying mechanism, we found that while the expression of cyclin-dependent kinase (CDK) inhibitor P21, one of the most commonly induced genes by HDACi [29], was significantly induced in all four pancreatic cancer cell lines upon treatment with VPA; however, the protein levels of cyclin D1 were only significantly decreased in the EGFR/ErbB2/ErbB3-coexpressing HPAF-II and MPanc96 cells (Fig. 1d).

In addition to growth inhibition, we then studied whether VPA might induce apoptosis in pancreatic cancer cells. Western blot analyses revealed that treatment with VPA induced PARP cleavage and activation of caspase-3 evidenced by the increase of cleaved caspase-3 in the HPAF-II and MPanc96 cells dramatically (Fig. 1e and g), but only marginally promoted apoptosis in MiaPaca-2 and Panc-1 (data not shown), suggesting that VPA selectively induced caspase-dependent apoptosis in the EGFR/ErbB2/ErbB3-coexpressing pancreatic cancer cells. Furthermore, a quantitative apoptotic ELISA showed that the induction fold of apoptosis significantly increased with prolonged treatment or increased dose of VPA (Fig. 1f and h), suggesting that VPA induced apoptosis in the EGFR/ErbB2/ErbB3-coexpressing pancreatic cancer cells in both dose- and time-dependent manners. VPA-induced apoptosis in the EGFR/ErbB2/ErbB3-coexpressing pancreatic cancer cells was also confirmed by flow cytometry analysis (Additional file 2: Figure S1). Collectively, our results demonstrate that VPA selectively exerts anti-tumor activity in vitro through induction of growth inhibition as well as apoptosis in the EGFR/ErbB2/ErbB3-coexpressing pancreatic cancer cells.

### VPA treatment down-regulates expression of EGFR, ErbB2, as well as ErbB3 in pancreatic cancer cells

**Fig. 2:**
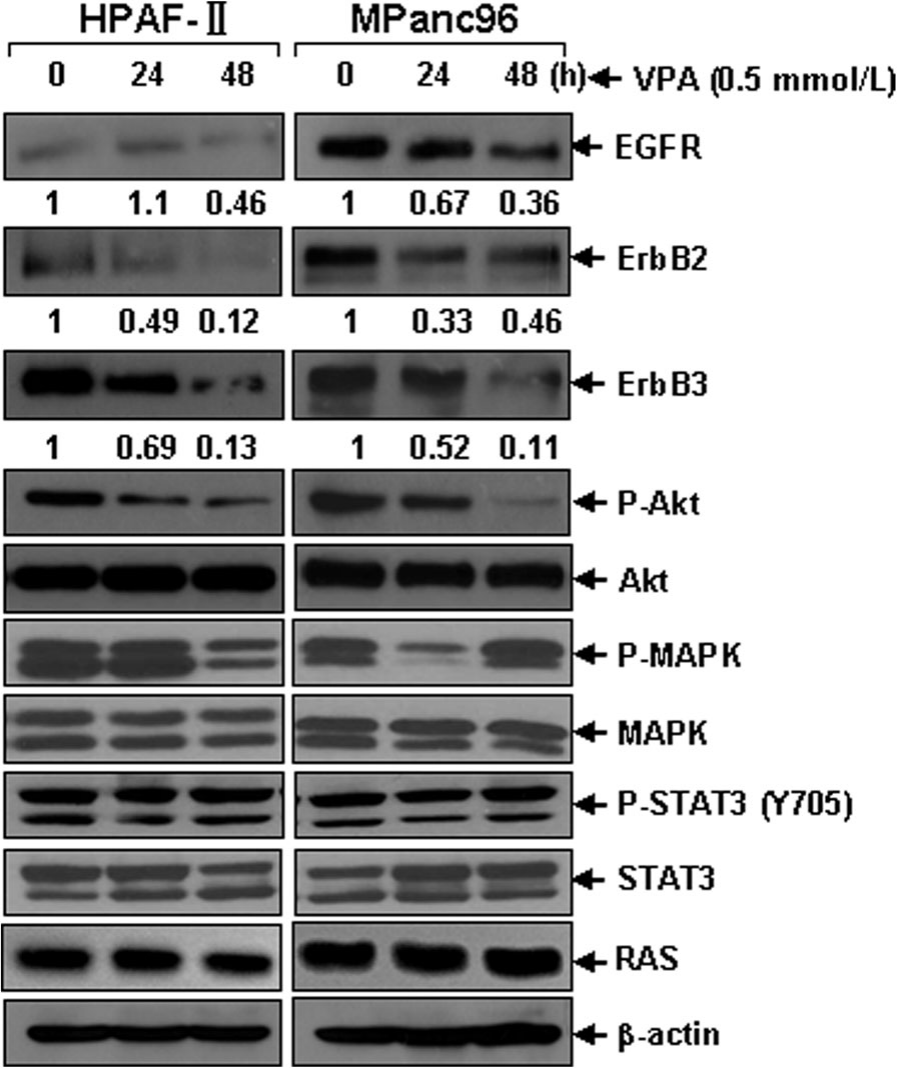
VPA significantly down-regulates the expression of ErbB family members EGFR, ErbB2, and ErbB3 and modulates the activation of downstream Akt and MAPK signalings. HPAF-II and MPanc96 cells untreated or treated with VPA (0.5 mmol/L) for 24 h or 48 h were harvested and subjected to western blot analyses with specific antibodies directed against EGFR, ErbB2, ErbB3, P-Akt, Akt, P-MAPK, MAPK, P-STAT3, STAT3, RAS or β-actin. The densitometry analyses of EGFR, ErbB2, and ErbB3 signals were shown underneath, and the arbitrary numbers indicate the intensities of each cell line relative to controls, defined as 1.0

ErbB signaling exerts a broad spectrum of tumor biological functions which play important roles in several hallmarks of cancer [30]. We previously showed that SNDX-275, a class I-selective HDACi, induced apoptosis in ErbB2-overexpressing breast cancer cells via down-regulation of both ErbB2 and ErbB3 [23]. In our attempt to determine the underlying mechanisms of anti-pancreatic cancer activity of VPA, the possible effect of VPA on expression of EGFR, ErbB2 and ErbB3 was investigated. As anticipated, upon treatment with VPA, the protein levels of EGFR, ErbB2 and ErbB3 in HPAF-II cells, and that of both EGFR and ErbB3 in MPanc96 cells were significantly reduced in a time-dependent manner (Fig. 2). Interestingly, while the expression of ErbB2 in MPanc96 cells decreased dramatically in a relative short time exposure to VPA, it recovered to some extent upon prolonged treatment with VPA (Fig. 2), which may reflect the happening of a feedback upregulation of ErbB2 in MPanc96 cells being similar to a phenomenon reported in breast cancer cells previously [31, 32]. Consequently, down-regulation of the ErbB family members resulted in significant inactivation of downstream Akt and MAPK signalings but not Stat3 signaling evidenced by decreased levels of both P-Akt and P-MAPK in HPAF-II and MPanc96 cells treated with VPA (Fig. 2). However, the expression of RAS remained unaltered. Thus, VPA treatment simultaneously down-regulates expression of EGFR, ErbB2, and ErbB3 in the ErbB family members-coexpressing pancreatic cancer cells, which in turn results in induction of cell growth inhibition as well as apoptosis.

### Specific knockdown of ErbB3 expression or blocking of downstream Akt signaling enhances VPA-induced apoptosis in pancreatic cancer cells

**Fig. 3:**
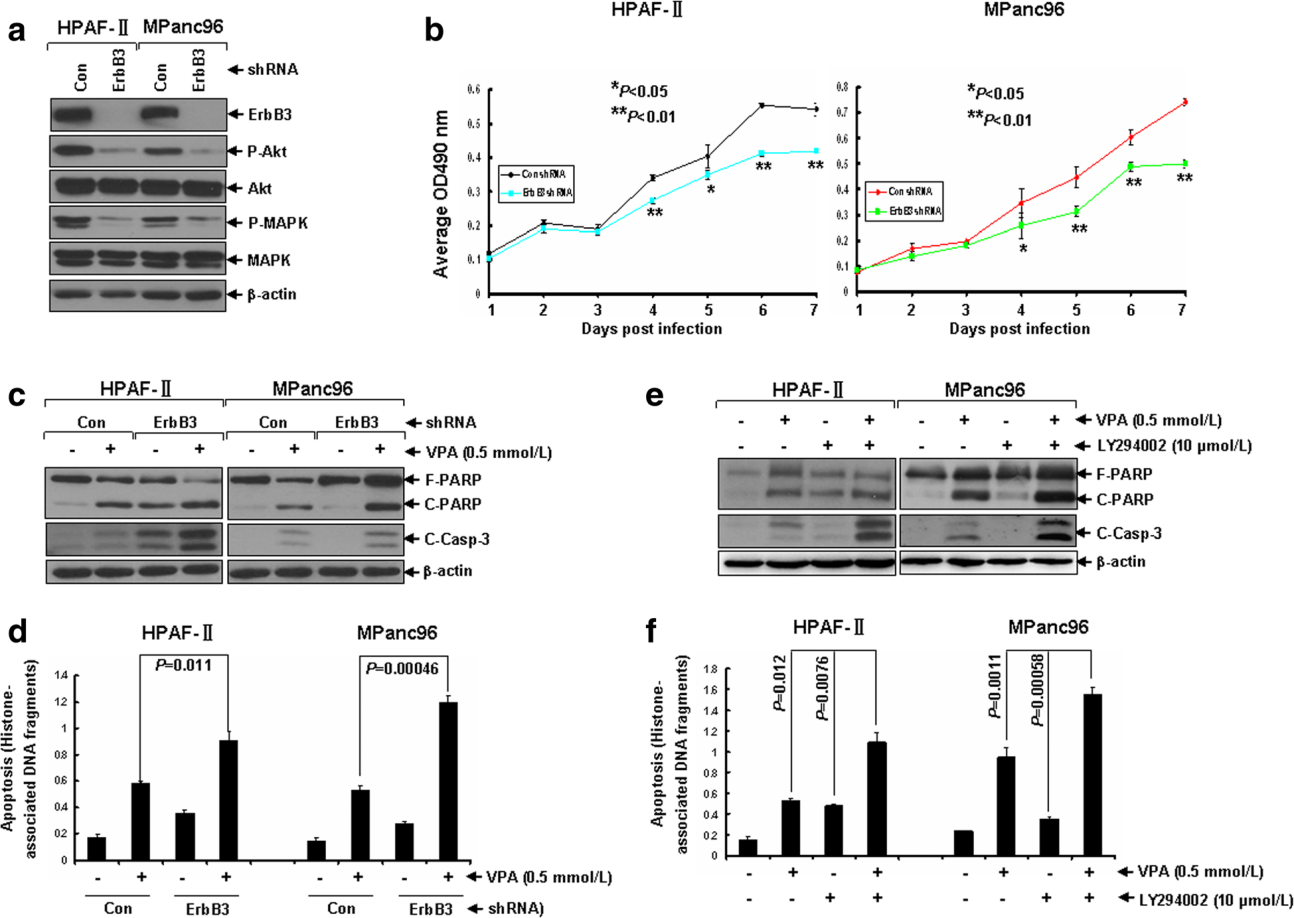
Specific knockdown of ErbB3 expression or blocking of its downstream signaling potentiates VPA-induced apoptosis in pancreatic cancer cells. **a** HPAF-II and MPanc96 cells were infected with ConshRNA- or ErbB3shRNA-expressing letiviruses. After 24 h, infected cells were subjected to selection with puromycin (1 μg/mL) for additional 24 h. Cells were then harvested and subjected to western blot analyses with specific antibodies directed against ErbB3, P-Akt, Akt, P-MAPK, MAPK or β-actin. **b** Growth curves of HPAF-II and MPanc96 cells without or with specific knockdown of ErbB3 expression. **c**, **d** HPAF-II and MPanc96 cells without or with transient knockdown of ErbB3 expression were cultured with RPMI1640 (0.5% FBS) in the presence or absence of VPA (0.5 mmol/L) for 24 h. Cells were harvested and subjected to antibodies directed against PARP, Cleaved-Caspase-3 or β-actin (**c**), or apoptotic ELISA (**d**). **e**, **f** HPAF-II and MPanc96 cells untreated or treated with either VPA (0.5 mmol/L) or LY294002 (10 μmol/L) alone, or combinations of VPA and LY294002 for 24 h were collected and subjected to western blot analyses with specific antibodies directed against PARP, C-Casp-3 or β-actin (**e**) or apoptotic-ELISA (**f**). *Bars*, S.D. Data show the representative of three independent experiments

Upon ligand binding, ErbB family members form homodimers or heterodimers to activate the downstream signaling to exert their biological functions. Among all possible heterodimers of ErbB family members, the ErbB2/ErbB3 heterodimers have been demonstrated to be the most prevalent and potent complexes [33]. In pancreatic cancer cells, the formation of EGFR/ErbB3 heterodimers are also evidenced to influence the pancreatic cancer cells’ sensitivity to erlotinib [34]. Given the fact that both the mRNA and protein expression of ErbB3 is comparative high in both HPAF-II and MPanc96 cells (Additional file 3: Figure S2 and Fig. 2), we then investigated whether specific down-regulation of ErbB3 expression may have any enhanced anti-survival effects in combination with VPA. While specific knockdown of ErbB3 was achieved via an ErbB3shRNA-expressing letiviral system in both HPAF-II and MPanc96 cells, downstream Akt and MAPK signalings were inhibited dramatically in parallel (Fig. 3a). The proliferation of both HPAF-II and MPanc96 cells with decreased ErbB3 expression was significantly inhibited as compared (Fig. 3b). More importantly, as compared with either ErbB3 knockdown or VPA treatment alone, the combinations of ErbB3 knockdown and VPA treatment showed significantly enhanced effects on induction of apoptosis in both HPAF-II and MPanc96 cells evidenced by increased cleavages of PARP and caspase-3 (Fig. 3c). The increased fold changes of VPA-induced apoptosis by knockdown of ErbB3 in both HPAF-II and MPanc96 cells were further verified by a specific apoptotic ELISA assay (Fig. 3d). Consistently, specific blocking of downstream Akt signaling with inhibitor of PI3K (LY294002) resulted in sensitization of HPAF-II and MPanc96 cells to VPA-induced apoptosis significantly (Fig. 3e and f). Collectively, these data suggest that specific knockdown of ErbB3 expression may enhances VPA-induced apoptosis in pancreatic cancer cells via blockade of the downstream Akt signaling.

### Introduced exogenous expression of either ErbB2 or ErbB3 attenuates VPA-induced inhibition of cell proliferation as well as apoptosis in pancreatic cancer cells

**Fig. 4:**
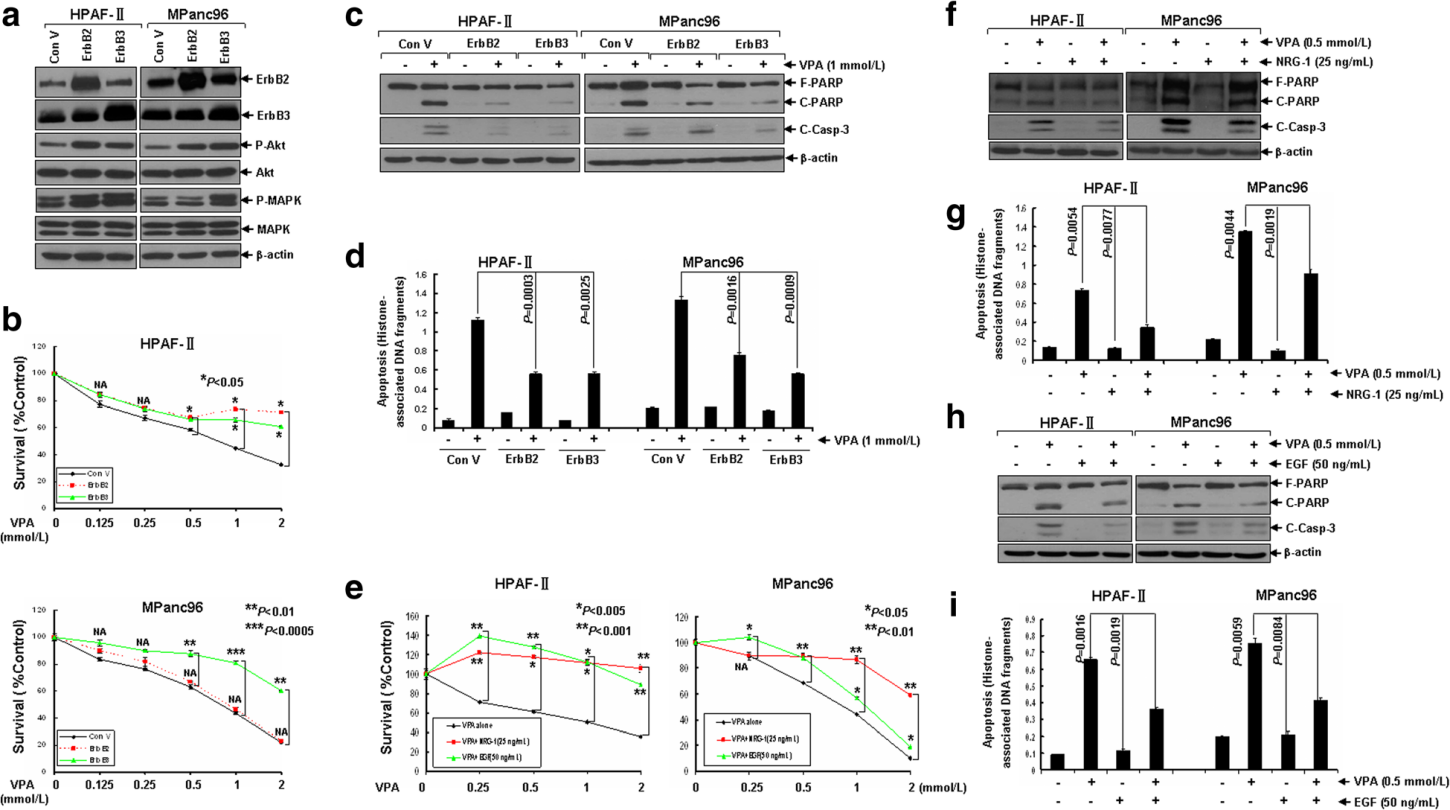
Elevated expression of either ErbB2 or ErbB3 or direct activation of their downstream signaling attenuates VPA-induced inhibition of cell proliferation/survival as well as apoptosis in pancreatic cancer cells. **a** Exogenous expression of either ErbB2 or ErbB3 was introduced via lentiviral expression system in HPAF-II and MPanc96 cells. Cells with stable resistance to Puromycin were harvested and subjected to western blot analyses with specific antibodies directed against ErbB2, ErbB3, P-Akt, Akt, P-MAPK, MAPK, or β-actin. **b** HPAF-II and MPanc96 cells without or with elevated expression of either ErbB2 or ErbB3 were plated onto 96-well plates with complete culture medium (RPMI1640, 10% FBS). After 24 h, the medium was replaced with control medium (fresh RPMI1640, 0.5% FBS) or same medium containing indicated concentrations of VPA for another 72 h incubation. The percentages of surviving cells from each treatment to controls, defined as 100% survival, were determined by reduction of MTS. **c**, **d** HPAF-II and MPanc96 cells without or with elevated expression of either ErbB2 or ErbB3 were treated with VPA (1 mmol/L) for 24 h. Cells were harvested and subjected to western blot analyses with specific antibodies directed against PARP, C-Casp-3 or β-actin (**c**), or apoptotic ELISA (**d**). **e** HPAF-II and MPanc96 cells were plated onto 96-well plates with complete culture medium (RPMI1640, 10% FBS). After 24 h, the medium was replaced with control medium (fresh RPMI1640, 0.5% FBS) or same medium containing indicated concentrations of either VPA alone, indicated concentrations of VPA in combination with NRG-1 (25 ng/mL), or indicated concentrations of VPA in combination with EGF (50 ng/mL) for another 72 h incubation. The percentages of surviving cells from each treatment to controls, defined as 100% survival, were determined by reduction of MTS. **f**, **g** HPAF-II and MPanc96 cells untreated or treated with either VPA (0.5 mmol/L) or NRG-1 (25 ng/mL) alone, or combinations of VPA and NRG-1 for 24 h were collected and subjected to western blot analyses with specific antibodies directed against PARP, C-Casp-3 or β-actin (**f**) or apoptotic-ELISA (**g**). **h**, **i** HPAF-II and MPanc96 cells untreated or treated with either VPA (0.5 mmol/L) or EGF (50 ng/mL) alone, or combinations of VPA and EGF for 24 h were collected and subjected to western blot analyses with specific antibodies directed against PARP, C-Casp-3 or β-actin (**h**) or apoptotic-ELISA (**i**). *Bars*, S.D. Data show the representative of three independent experiments

Next, we sought to determine whether down-regulation of ErbB family members is required for VPA-induced growth inhibition and apoptosis in pancreatic cancer cells. Exogenous expression of either ErbB2 or ErbB3 was introduced via lentiviral expression system in HPAF-II and MPanc96 cells (Fig. 4a). Overexpression of ErbB3 significantly abrogated VPA-mediated anti-proliferation/anti-survival effects in both HPAF-II and MPanc96 cells, whereas ectopic expression of ErbB2 showed similar effects only in HPAF-II but not MPanc96 cells (Fig. 4b). Furthermore, exogenous expression of either ErbB2 or ErbB3 decreased the levels of cleaved PARP and caspase-3 induced by VPA (Fig. 4c). Significantly reduced VPA-induced apoptosis in pancreatic cancer cells with exogenous expression of either ErbB2 or ErbB3 were further demonstrated by a specific apoptotic ELISA assay (Fig. 4d). Consistently, direct activation of either ErbB3 or EGFR signaling with specific ligands (NRG-1 and EGF, respectively) also resulted in significant abrogation of anti-pancreatic cancer activity of VPA (Fig. 4e-i). Collectively, specific activation or forced overexpression of either ErbB2 or ErbB3 attenuates VPA-induced apoptosis and inhibition of cell proliferation/survival in pancreatic cancer cells via constitutive activation of downstream Akt and MAPK signaling pathways.

### VPA treatment induces expression of ErbB family members-targeting microRNAs in pancreatic cancer cells without altering mRNAs levels of EGFR, ErbB2, and ErbB3

**Fig. 5:**
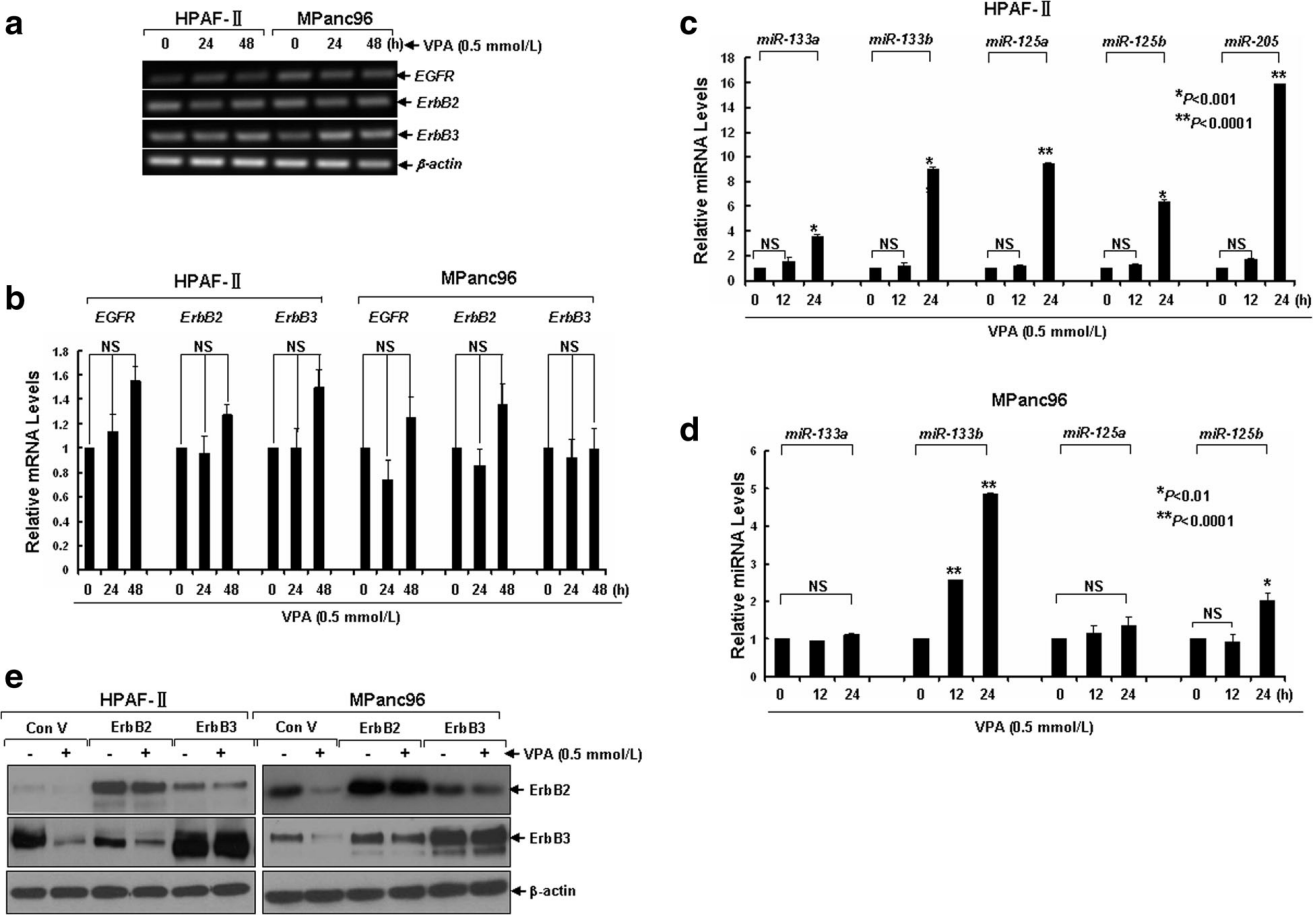
VPA induces expression of ErbB family members-targeting microRNAs in pancreatic cancer cells without altering mRNAs levels of *EGFR*, *ErbB2*, and *ErbB3*, which results in down-regulation of endogenous but not exogenous ErbB2 and ErbB3. **a**, **b** HPAF-II and MPanc96 cells untreated or treated with VPA (0.5 mmol/L) for 24 h or 48 h were harvested for total RNA preparation and subjected for analysis of mRNAs expression levels of *EGFR*, *ErbB2*, and *ErbB3* with conventional (**a**) or quantitative RT-PCR (**b**). **c**, **d** HPAF-II and MPanc96 cells untreated or treated with VPA (0.5 mmol/L) for 24 h or 48 h were collected and subjected to total RNA extraction, inclusive of the small RNA fraction. The expression levels of miR-133a, miR-133b, miR-125a, miR-125b, and miR-205 were measured by qRT-PCR using TaqMan miRNA assays. All results were normalized with the internal control RNU6B. *Bars*, S.D. All data show the representative of three independent experiments. **e** HPAF-II and MPanc96 cells without or with elevated expression of either ErbB2 or ErbB3 were treated with VPA (0.5 mmol/L) for 24 h. Cells were harvested and subjected to western blot analyses with specific antibodies directed against ErbB2, ErbB3, or β-actin

To investigate the molecular mechanism by which VPA down-regulated EGFR, ErbB2, and ErbB3 in pancreatic cancer cells, we next explored whether treatment with VPA might modulate *EGFR*, *ErbB2*, and *ErbB3* mRNA levels. Conventional reverse transcriptase-PCR assay revealed that treatment with VPA did not show significant effect on the mRNA levels of *EGFR*, *ErbB2*, and *ErbB3* in pancreatic cancer HPAF-II and MPanc96 cells (Fig. 5a). Similar results were further confirmed by the qRT-PCR assays (Fig. 5b). These results suggested that VPA might down-regulate EGFR, ErbB2, and ErbB3 through a transcription-independent mechanism.

Currently, miRNA emerges as an important mediator in post-transcriptional regulation of expression of gene of interest (GOI). Since we previously clarified that down-regulation of ErbB2 and ErbB3 in breast cancer cells was attributed to induction of ErbB2- and ErbB3-targeting miRNAs (miR-125a, miR-125b, and miR-205) by HDACi entinostat [24], we then asked whether similar mechanisms might account for VPA-induced down-regulation of the ErbB family members in pancreatic cancer cells. While the expression levels of miR-125a, miR-125b, and miR-205 in HPAF-II cells were markedly elevated by VPA, that of miR-125b in MPanc96 cells was also evidenced to be increased by VPA by a qRT-PCR assay (Fig. 5c and d). However, treatment with VPA did not alter the expression of miR-125a in MPanc96 cells, in which the expression of miR-205 was undetectable (data not shown). As both miR-133a and miR-133b had been reported to target EGFR by several studies [35–37], they were then chosen to be candidates of miRNAs targeting EGFR whose expression might be influenced by VPA. Our qRT-PCR analysis revealed that upon treatment with VPA, the expression of miR-133a was slightly increased in HPAF-II but remained unaltered in MPanc96 cells. Meanwhile, the expression of miR-133b was significantly induced in both HPAF-II and MPanc96 cells by VPA (Fig. 5d and d). Interestingly, VPA was demonstrated to specifically reduce the protein levels of endogenous but not exogenous ErbB2 and ErbB3 (Fig. 5e). Given the fact that as compared, transcripts derived from exogenous genes contain coding sequence of GOI alone but lacking of original 5′-UTR and 3′-UTR, two regions with binding sites of most miRNAs, which means that they are with less chance to be targeted by miRNAs. Taken together, our data demonstrate that down-regulation of EGFR, ErbB2, and ErbB3 in HPAF-II and MPanc96 cells by VPA is more likely due to induction of the miRNAs that target the *erbB* mRNAs.

### VPA inhibits tumor growth in a xenograft model of EGFR/ErbB2/ErbB3-coexpressing pancreatic cancer

**Fig. 6:**
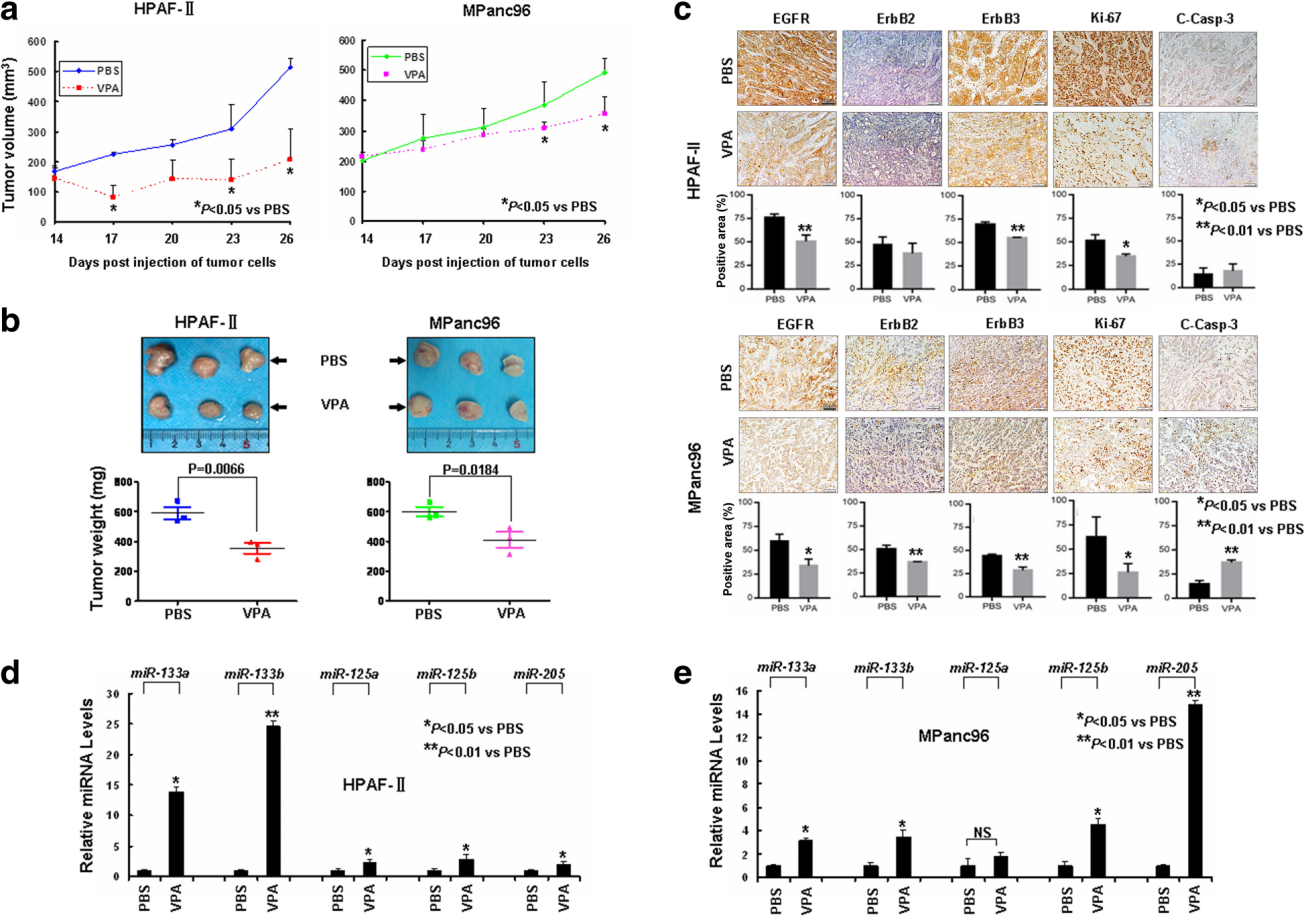
VPA inhibits in vivo tumor growth of EGFR/ErbB2/ErbB3-coexpressing pancreatic cancer cells in a murine model. HPAF-II and MPanc96 cells were subcutaneously injected into nude mice to establish tumor xenografts. The tumor-bearing mice (*n* = 3) received intraperitoneal injections of either PBS or VPA (500 mg/kg) as described in the Materials and Methods. After treatments, the mice were euthanized at day 26 post injection of tumor cells and all tumors were excised for IHC and qRT-PCR analysis. **a** The graphs show the tumor growth curves. *Bars*, S.D. **b** Data show the excised tumors and tumor weights from each treatment group. **c** Data show the representative images of the immunostainging of EGFR, ErbB2, ErbB3, Ki-67, and C-Casp-3. The IHC slides were observed by two independent personnel. The tumor cells with positive staining of EGFR, ErbB2, ErbB3, Ki-67, or C-Casp-3 were counted from three randomly selected areas in each slide. The three areas were first identified by scanning the entire slide at × 100 magnification, and then the positive-stained cells were counted at × 200 magnification using an Olympus BX40 microscope. The bar graphs show the average percent of positive staining cells in each field. Bars represent S.D. *P < 0.05 versus PBS control. **P < 0.01 versus PBS control. **d**, **e** qRT-PCR analysis of the expression levels of miR-133a, miR-133b, miR-125a, miR-125b, and miR-205 in xenograft tumors. All results were normalized with the internal control RNU6B. *Bars*, S.D. All data show the representative of three independent experiments

To determine whether VPA holds in vivo anti-tumor activity, we took advantage of tumor xenograft models established from HPAF-II and MPanc96 cells. When tumor volume reach ~ 150 mm^3^, the tumor bearing mice were treated with either PBS or VPA (500 mg/kg) daily for consecutive 12 days. Our data showed that treatment with VPA significantly inhibited tumor growth as compared with PBS (Fig. 6a and b). To examine the effects of VPA on cell proliferation and apoptosis in vivo, the tumor tissues were subjected to IHC analyses of Ki-67 and cleaved caspase-3. While VPA dramatically reduced Ki-67-staining cells in both xenograft models, it significantly increased cleaved caspase-3 only in tumor xenograft model derived from MPanc96 cells (Fig. 6c). Moreover, VPA held potential of in vivo induction of ErbB family members-targeting microRNAs and down-regulation of ErbB family members in xenograft tumors derived from both HPAF-II and MPanc96 cells (Fig. 6c, d and e), which was consistent with our in vitro findings (Figs. 3 and 5c and d). Thus, our in vivo data further confirmed that VPA exhibits anti-tumor activity selectively against EGFR/ErbB2/ErbB3-coexpressing pancreatic cancer likely via induction of miRNAs that target the *erbB* mRNAs.

## Discussion

Deregulated ErbB signalling has long been recognized as a key player in tumorigenesis of many solid tumors including pancreatic cancer [38]. Consequently, strategies of ErbB-targeting therapy have been extensively studied, among which agents directed against the EGFR are the best studied targeted agents in pancreatic cancer [9]. However, patients with pancreatic cancer benefit little from current existed therapies targeting EGFR [39, 40], which indicating that it is insufficient to overcome this disease by targeting EGFR alone. Hence, simultaneous targeting of coexpressing ErbB family members is of particular interest in developing new strategy against pancreatic cancer. In fact, several studies had showed that combined targeting of EGFR and ErbB2 did result in enhanced efficacy in treatment of pancreatic cancer [19, 20]. In addition to EGFR and ErbB2, their kin ErbB3 is emerging as an attractive druggable target in pancreatic cancer [41–43]. However, no ErbB3-targeted therapy has been approved for cancer treatment to date. In our attempt to seek novel strategy to treat pancreatic cancer, we found that VPA exhibits a promising anti-tumor activity selectively against EGFR/ErbB2/ErbB3-coexpressing pancreatic cancer. VPA not only preferentially inhibits proliferation/survival of aforementioned subtype of pancreatic cancer cells in a dose-dependent manner (Fig. 1), but also potently induces caspase-dependent apoptosis in EGFR/ErbB2/ErbB3-coexpressing pancreatic cancer cells within its clinically achievable range [40~100 mg/L (0.24~0.6 mmol/L)] (Fig. 1). Mechanistically, VPA treatment was evidenced to result in significant inactivation of Akt and MAPK signalings via simultaneous down-regulation of EGFR, ErbB2, as well as ErbB3 in pancreatic cancer cells (Fig. 2). More importantly, the in vivo anti-tumor activity of VPA against ErbB family members-coexpressing pancreatic cancer was further confirmed. These findings suggest that cooperation between ErbB family members is requisite in regulating cell proliferation and survival in pancreatic cancer, thus simultaneously targeting multiple ErbB family members should be more potent than targeting individual alone. In consideration of this, VPA may be an excellent candidate drug against pancreatic cancer with a strong activity of triple-targeting EGFR, ErbB2, and ErbB3.

The anti-tumor activity of VPA has been investigated whether alone or in combination with conventional therapeutic such as epirubicin [44]. In term of molecular mechanism, VPA was showed to synergize with fluoropyrimidines in against breast and colorectal cancer via downregulating thymidylate synthase and upregulating thymidine phosphorylase [45–47]. Recently, VPA was also evidenced to synergize with capecitabine and radiotherapy in colorectal cancer via modulation of both wild-type and mutant p53 [48], and both VPA and Trichostatin A (TSA) were shown to induce pancreatic cancer cell apoptosis and autophagy by Gilardini Montani et al. [49]. Interestingly, a study by La Noce et al. indicated that VPA could induce an increased expression of stem markers including CD133, OCT4, SOX2 and NANOG in osteosarcoma cell lines, and an increased ability in sarcospheres and colonies formation efficiency, suggesting that HDAC2 is a key factor regulating both cancer stem cells (CSCs) phenotype and in vivo cancer growth [50]. We reported here that VPA-induced simultaneous down-regulation of EGFR, ErbB2, and ErbB3 in pancreatic cancer cells could be mainly attributed to induction of ErbB family members-targeting miRNAs both in vitro and in vivo (Figs. 5 and 6). Interestingly, upon long term treatment with VPA, silenced miR-205 was significantly induced in tumor xenograft derived from MPanc96 cells (Fig. 6e). This is similar to the mechanism underlying down-regulation of ErbB2 and ErbB3 by entinostat we found previously [23, 24]. Nonetheless, the molecular mechanism by which VPA induces expression of ErbB family members-targeting miRNAs awaits further investigation. Our current study adds new knowledge to the understanding of mechanism of VPA against pancreatic cancer.

## Conclusions

We demonstrated that VPA inhibits proliferation/survival of pancreatic cancer cells as well as induces caspase-dependent apoptosis selectively in EGFR/ErbB2/ErbB3-coexpressing pancreatic cancer within its clinically achievable range. Mechanistic investigations revealed that VPA simultaneously down-regulates EGFR, ErbB2 and ErbB3 likely via induction of several critical miRNAs in pancreatic cancer cells, and thereby results in inactivation of downstream Akt and MAPK signaling pathways and induces cell apoptosis and growth arrest. Our data strongly suggest that VPA may be added to the treatment regimens for pancreatic cancer patients with co-overexpression of the ErbB family members.

## Additional files

Additional file 1: **Table S1.** Primers used for RT-PCR and qRT-PCR. (DOC 27 kb)

Additional file 2: **Figure S1.** Pro-apoptotic effect induced by VPA in EGFR/ErbB2/ErbB3-coexpressing pancreatic cancer cells. HPAF-II and MPanc96 cells untreated or treated with VPA (0.5 mmol/L) for 24 h or 48 h were harvested and subjected to flow cytometry assay. a Representative results of flow cytometry analysis. b Apoptotic effect was evaluated by flow cytometry analysis upon propidiumiodide (PI) and annexin V-FITC co-staining. Data show the representative of three independent experiments. (TIF 929 kb)

Additional file 3: **Figure S2.** Relative mRNA levels of *EGFR*, *ErbB2*, and *ErbB3* in pancreatic cancer cells. Parental HPAF-II and MPanc96 cells were harvested for total RNA preparation and subjected for analysis of mRNAs expression levels of *EGFR*, *ErbB2*, and *ErbB3* with quantitative RT-PCR. *Bars*, S.D. Data show the representative of three independent experiments. (TIF 121 kb)

## Abbreviations

EGFR: Epidermal growth factor receptor
ELISA: Enzyme-linked immunosorbent assay
ErbB: Erythroblastic leukemia viral oncogene homolog
GOI: Gene of interest
HDAC: Histone deacetylase
HDACi: HDAC inhibitor
IHC: Immunohistochemistry
MAPK: Mitogen-activated protein kinase
MTS: 3-(4,5-dimethylthiazol-2-yl)-5-(3-carboxymethoxyphenyl)-2-(4-sulfophenyl)-2H-tetrazolium, inner salt
PARP: Poly (ADP-ribose) polymerase
PI-3 K: Phosphoinositide 3-kinase
qRT-PCR: Real-time quantitative reverse transcriptase PCR
RTKs: Receptor tyrosine kinases
UTR: Untranslated region
VPA: Valproic acid
