# Supplementary material for: Valproic acid exhibits anti-tumor activity selectively against EGFR/ErbB2/ErbB3-coexpressing pancreatic cancer via induction of ErbB family members-targeting microRNAs
Source: J Exp Clin Cancer Res. 2019 Apr 8;38:150. doi: 10.1186/s13046-019-1160-9 (PMC6454766; doi:10.1186/s13046-019-1160-9)
Supplement: Supplementary file 1 — Table S1. Primers used for RT-PCR and qRT-PCR. (DOC 27 kb) [file 13046_2019_1160_MOESM1_ESM.doc]

**Supplemental Table S1: Primers used for RT-PCR and qRT-PCR**

| **Gene** | **Name** | **Sequence** |
| --- | --- | --- |
| *EGFR*  *ErbB2*  *ErbB3*  *β-actin* | hEGFR_F  hEGFR_R  hErbB2_F  hEerB2_R  hErbB3_F  hErbB3_R  hβ-actin _F  hβ-actin _R | 5’-gaatgcatttgccaagtcct-3’  5’-agctttgcagcccatttcta-3’  5’-aaaggcccaagactctctcc-3’  5’-actctgggttctctgccgta-3’  5’-caagttcccttgaggagctg-3’  5’-catctcgttgccgattcata-3’  5’-AGAGCTACGAGCTGCCTGAC-3’  5’-AGCACTGTGTTGGCGTACAG-3’ |
