# Supplementary figures and images for: Valproic acid exhibits anti-tumor activity selectively against EGFR/ErbB2/ErbB3-coexpressing pancreatic cancer via induction of ErbB family members-targeting microRNAs
Source: J Exp Clin Cancer Res. 2019 Apr 8;38:150. doi: 10.1186/s13046-019-1160-9 (PMC6454766; doi:10.1186/s13046-019-1160-9)

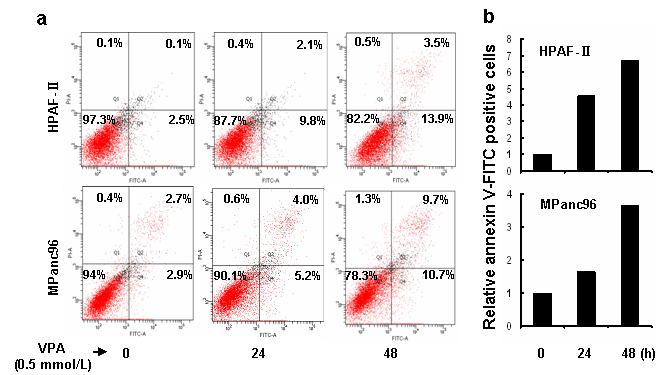

Supplement: Supplementary file 2 — Figure S1. Pro-apoptotic effect induced by VPA in EGFR/ErbB2/ErbB3-coexpressing pancreatic cancer cells. HPAF-II and MPanc96 cells untreated or treated with VPA (0.5 mmol/L) for 24 h or 48 h were harvested and subjected to flow cytometry assay. a Representative results of flow cytometry analysis. b Apoptotic effect was evaluated by flow cytometry analysis upon propidiumiodide (PI) and annexin V-FITC co-staining. Data show the representative of three independent experiments. (TIF 929 kb) [file 13046_2019_1160_MOESM2_ESM.tif]

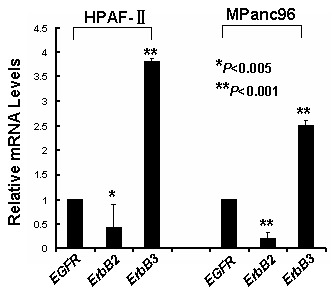

Supplement: Supplementary file 3 — Figure S2. Relative mRNA levels of EGFR, ErbB2, and ErbB3 in pancreatic cancer cells. Parental HPAF-II and MPanc96 cells were harvested for total RNA preparation and subjected for analysis of mRNAs expression levels of EGFR, ErbB2, and ErbB3 with quantitative RT-PCR. Bars, S.D. Data show the representative of three independent experiments. (TIF 121 kb) [file 13046_2019_1160_MOESM3_ESM.tif]
